# Clinical and epidemiological characteristics of *Helicobacter pylori* infection among patients in specialty outpatient clinics at Faculdade de Medicina ABC

**DOI:** 10.1590/S1516-31802007000500012

**Published:** 2007-09-02

**Authors:** Ethel Zimberg Chehter, Fabio Luiz Maximiano, Leonardo Fernando Ferrari Nogueira, Fernando Antônio Blandi, Ricardo Eugênio Mariani Burdelis, Maurício Fullep Ferreira Xavier, Fernando Fonseca, Wilson Roberto Catapani

*Helicobacter pylori (H. pylori)* affects 60% of the worldwide population.^1^ It is recognized as the most important agent for chronic gastritis^1^ and is present in 90% of duodenal ulcers and 70% of gastric ulcers.^2^ It is also a cofactor for gastric cancer and mucosa-associated lymphoid tissue (MALT) lymphoma.^1^

Two infection patterns can be identified: in developed countries, the prevalence increases with age,^3^ while in developing countries, the young population is already infected, due to poor living conditions and low schooling levels.^4^

The ABC São Paulo region is an area with concentrations of industrial activity, with a high mean human development index (HDI) of 0.819.^5^ Faculdade de Medicina do ABC (FMABC) is located in this region and therefore the population chosen for our study consisted of patients attending specialty outpatient clinics at FMABC. The aims of our study were to determine the *H. pylori* infection rate in this population, find associations between infection rates and socioeconomic conditions, and try to establish possible associated risk factors.

This cross-sectional screening study was carried out from August 2002 to May 2003 in the specialty outpatient clinics of this institution, and the patients who agreed to participate filled out a questionnaire and consented to having a sample of blood drawn to perform the *H. pylori* serological test.

The questionnaire covered demographic data such as gender, age, race, place of birth and occupation; symptoms (pyrosis, epigastric pain or dyspepsia); presence of ulcers; use of tobacco, alcohol and non-steroidal anti-inflammatory drugs (NSAIDs); and also socioeconomic and cultural characteristics such as schooling level, house ownership, basic sanitation, number of rooms in the home and number of inhabitants per household.

Enzyme immunoassay serology was used to detect anti-*H. pylori* immunoglobulin-G (IgG) antibodies using the Accubind^TM^ *H. pylori* IgG kit (Monobind, Inc., United States). According to the manufacturer, this kit presents 98.7% sensitivity and 97.0% specificity.

The results obtained were subjected to statistical analysis. The Mann-Whitney test was applied to the study variables, using a significance level of 5%. Multiple logistic regression was used, with 95% confidence intervals, to ascertain whether the variables presented any associations with the serological results.

The study included 513 patients, who were mostly female (68.8%), had a mean age of 50.6 years (range: 18-87), lived in urban areas, had good socioeconomic status, but had low schooling levels. *H. pylori*-positive serological results (IgG) were found in 72.5% of the study population.

Statistical analysis did not reveal any associations between infection and gender, alcohol use or tobacco use. However, a higher infection risk was found among non-whites, and there was an association between positive serology and low schooling levels.

Even though the study population was from the ABC São Paulo region, which is known to have a high socioeconomic level, with good rates of basic sanitation and few overcrowded households, thus indicating close similarity to the characteristics of developed countries, the infection rate due to *H. pylori* found here was similar to that of developing countries. In this respect, our findings differ from what is reported in the worldwide literature.^3^

This difference might be explained by the low schooling levels found, thus suggesting that hygiene and dietary principles are not well known, which could lead to high infection rates by this bacterium.
